# Exploring the relationship between NHHR and the degree of coronary artery stenosis in patients with acute coronary syndromes

**DOI:** 10.1186/s12872-025-05066-z

**Published:** 2025-08-07

**Authors:** Zhenkun Yang, Yuanjie Li, Mingjuan Yang, Yang Xu, Jia Yao, Kefan Wang, Yuxia Gao

**Affiliations:** 1 Department of Cardiology, Tianjin Medical University General Hospital, Tianjin, China; 2 Tianjin Research Institute of Anesthesiology, Department of Anesthesiology, Tianjin Medical University General Hospital, Tianjin, China

**Keywords:** ACS, NHHR, Gensini score, Lipid parameters, Nomogram

## Abstract

**Objective:**

This study explores the relationship between the non-high-density lipoprotein cholesterol to high-density lipoprotein cholesterol ratio (NHHR) and the severity of coronary artery stenosis in acute coronary syndrome (ACS) patients.

**Methods:**

We included patients who were first admitted to the cardiac intensive care unit of Tianjin Medical University General Hospital between July 1, 2022, and June 30, 2024, with a diagnosis of ACS. Coronary stenosis severity was assessed using the Gensini score (GS), with patients divided into high (≥ 68.5) and low (< 68.5) GS groups. Patients in the high GS group represented those with more severe coronary artery stenosis. General clinical data from the first admission were compared between the two groups. Logistic regression identified independent risk factors, and receiver operating characteristic (ROC) curves evaluated the predictive value of NHHR and other lipid parameters. C-statistics, calibration, and decision curves assessed the Nomogram’s predictive accuracy. Sensitivity analysis was performed to further validate the robustness of the results.

**Results:**

The study included 1,799 ACS patients, 907 with severe coronary artery lesions (GS ≥ 68.5). The median age was 71, and 72.9% were male. NHHR was significantly higher in the high GS group. Logistic regression showed NHHR as an independent risk factor for high GS (odds ratio, OR = 1.15, *P* = 0.001), with higher NHHR levels indicating greater risk. Compared to the first quartile (Q1), the third and fourth quartiles showed increased risk (OR = 1.36, *P* = 0.044; OR = 1.66, *P* = 0.002). Incorporating NHHR into the model for predicting severe coronary artery lesions (Model 1) increased the predictive value from 0.696 (95% CI: 0.674–0.717) to 0.703 (95% CI: 0.682–0.724).

**Conclusion:**

NHHR was an independent risk factor for severe coronary artery stenosis (GS ≥ 68.5) in ACS patients, with higher values linked to increased lesion severity. It outperformed traditional lipid parameters in predicting severity and improved the prediction model’s accuracy. Subgroup analysis showed stronger associations in high-risk populations, though further studies are needed to confirm its clinical utility.

**Supplementary Information:**

The online version contains supplementary material available at 10.1186/s12872-025-05066-z.

## Introduction

ACS is a serious cardiovascular disease group caused by acute myocardial ischemia [1]. It includes ST-elevation myocardial infarction (STEMI) (30%) and non-ST-elevation acute coronary syndrome (70%), the latter including non-ST-elevation myocardial infarction (NSTEMI) and unstable angina (UA). Over 7 million people worldwide are diagnosed with ACS annually [2], with a higher incidence observed in populations aged 65 and older, as well as in rural areas and some low- and middle-income countries [2, 3].

Dyslipidemia is one of the major risk factors for atherosclerosis, and plaque rupture or erosion is the core pathological process of ACS [4]. Elevated lipoprotein(a) levels have been increasingly recognized as an independent and causal risk factor for atherosclerotic cardiovascular disease, including myocardial infarction and stroke [5]. NHHR is an important indicator used in cardiovascular research to assess the risk of atherosclerosis. It includes all lipoproteins that contribute to atherosclerosis, such as low-density lipoprotein cholesterol (LDL), very low-density lipoprotein cholesterol, and high-density lipoprotein cholesterol (HDL-C), which counters atherosclerosis [6]. A higher NHHR reflects excessive accumulation of atherosclerotic lipids in coronary arteries, thus exacerbating coronary artery disease [7]. Compared to single lipid indicators like LDL-C or HDL-C, NHHR is considered a stronger predictor of cardiovascular events [8]. This is because NHHR reflects the balance between atherosclerotic lipoproteins and anti-atherosclerotic lipoproteins, rather than just the abnormality of one specific type of lipoprotein.

The GS is a system used to evaluate the severity of coronary artery disease, primarily used for quantifying the results of coronary angiography (CAG) to reflect the degree of coronary stenosis and the extent of coronary artery lesions. The higher the score, the greater the degree of coronary stenosis and the more widespread the coronary artery disease [9]. Some new guidelines indicate that ACS patients with multi-vessel coronary disease have an extremely high risk of mortality and poor prognosis [10]. Data from the United States show that patients with coronary multi-vessel diffuse disease face significantly higher risks of myocardial infarction (MI) and death within one year [11]. The GS provides a more comprehensive understanding of the severity of coronary artery lesions in ACS patients, helping with the assessment of the disease and prediction of future cardiovascular events, thus guiding more targeted treatment strategies. As a simple and readily available lipid parameter, NHHR can assist clinicians in predicting the severity of coronary artery disease and inferring the level of the GS. NHHR has been shown to be related to various diseases, such as carotid atherosclerosis and plaque stability [12], metabolic syndrome [13], diabetes prevalence [14], depression risk in U.S. adults [15], kidney stone occurrence and recurrence [16], adult sleep disorders [17], periodontitis prevalence [18], and abdominal aortic aneurysm [19].

ACS is one of the most common conditions requiring urgent cardiac care [20]. Although primary prevention has improved, the incidence and related mortality of ACS under intensive care remain high [1]. Identifying patients at extremely high risk is crucial for saving lives, improving the quality of life of patients, and reducing the burden on the healthcare system. Currently, there is no established relationship between NHHR and the GS in ACS patients. The aim of our study is to explore the role of NHHR in the GS of ACS patients and further understand the relationship between various lipid parameters and the GS, to establish a prediction model for predicting more severe coronary lesions.

## Methods

### Study population

**Fig. 1:**
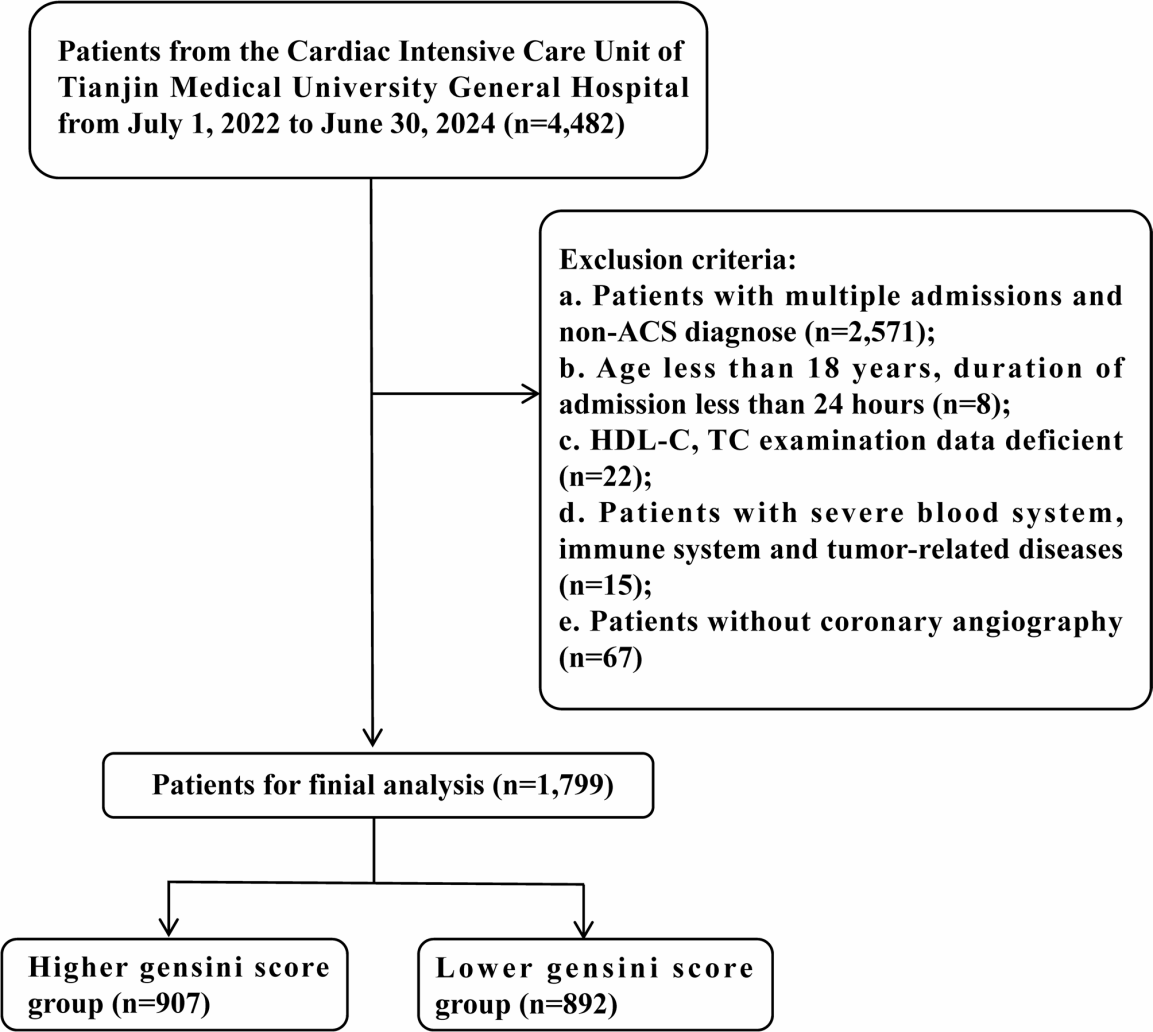
Flowchart. Abbreviations: ACS, acute coronary syndromes; HDL-C, high-density lipoprotein cholesterol; TC, triglyceride

Patients admitted to the coronary care unit (CCU) of Tianjin Medical University General Hospital from July 1, 2022, to June 30, 2024, were selected, and their electronic medical records were retrieved. A total of 1799 patients were included (Fig. 1). Inclusion criteria: (a) First-time admission to the CCU of Tianjin Medical University General Hospital; (b) Meeting the diagnostic criteria for ACS based on symptoms, electrocardiographic findings, myocardial injury markers, and relevant clinical data, in accordance with 2023 ESC Guidelines for the Management of Acute Coronary Syndromes Diagnostic Criteria [21]; (c) Complete case data. Exclusion criteria: (a) Age < 18 years; (b) Hospitalization duration < 24 h; (c) No coronary angiography performed; (d) Missing HDL-C or total cholesterol data; (e) Presence of hematologic, immune system, or tumor-related diseases.

The study was approved by the Ethics Committee of the General Hospital of Tianjin Medical University in accordance with the Declaration of Helsinki (Ethics approval number: IRB2024-YX-138-01). As the study was retrospective, informed consent was waived.

### Data collection

General patient information collected: hospital admission number, admission date, discharge date, age, gender, height, weight, smoking history, alcohol consumption history; blood pressure on the day of admission; Medical history: previous myocardial infarction, status post-coronary artery stent implantation, status post-coronary artery bypass grafting, hypertension, type 2 diabetes, atrial fibrillation, heart failure, cerebrovascular disease, renal insufficiency, hepatic insufficiency; Echocardiography results: left ventricular ejection fraction (LVEF), left ventricular end-diastolic diameter (LVEDD); Medications on admission: aspirin, clopidogrel, ticagrelor, rosuvastatin, atorvastatin, proprotein convertase subtilisin/kexin type 9 inhibitors (PCSK9i), sodium-glucose cotransporter 2 inhibitors (SGLT2i), GLP-1 receptor agonists (GLP-1RA). First fasting lab tests on admission: apolipoprotein A1 (ApoA1), apolipoprotein B (ApoB), lipoprotein(a) [Lp(a)], triglycerides (TG), LDL-C, HDL-C, total cholesterol (TC), D-Dimer, brain natriuretic peptide (BNP), fasting blood glucose (FBG), glycated hemoglobin (HbA1c), uric acid, creatinine, albumin, hypersensitive C-reactive protein (hs-CRP), complete blood count, etc. Body mass index (BMI) = weight/height² (kg/m²); mean arterial pressure (MAP) = 1/3 systolic pressure + 2/3 diastolic pressure. Non-HDL-C = TC - HDL-C, NHHR = Non-HDL-C/HDL-C.

### GS definition

All patients underwent percutaneous coronary angiography in the catheterization lab of Tianjin Medical University General Hospital, with two experienced interventional cardiologists analyzing and treating the target vessels. In cases of discrepancy, a consensus was reached after joint review. The procedural information for each patient’s current admission, including the location of coronary lesions and the degree of vessel stenosis, was collected. Detailed surgical records were obtained, and the GS was calculated. Specifically, coronary artery stenoses were graded based on the degree of luminal narrowing and assigned a severity score (e.g., 1 for 25% narrowing, 2 for 50%, 4 for 75%, 8 for 90%, 16 for 99%, and 32 for total occlusion), which was then multiplied by a weighting factor reflecting the anatomical location and significance of the lesion (Supplementary Table 1).

### Grouping definitions

Patients were categorized into two groups based on their median GS, i.e., high GS group (≥ 68.5), and low GS group (< 68.5). Patients were categorized into four groups based on their NHHR quartiles, i.e., Q1 (NHHR ≤ 2.75), Q2 (2.75 < NHHR ≤ 3.59), Q3 (3.59 < NHHR ≤ 4.51), and Q4 (NHHR > 4.51).

#### Statistical analysis

The Shapiro-Wilk test was used to assess the normality of quantitative data. Normally distributed data were expressed as mean ± standard deviation (Mean ± SD), while non-normally distributed data were expressed as median and interquartile range [M (Q1, Q3)]. Differences between groups were compared using the independent samples t-test or the Kruskal-Wallis rank-sum test. Categorical data were expressed as frequency and percentage n (%), and differences between groups were compared using the chi-square test or Fisher’s exact test. Spearman rank correlation coefficients were used to analyze the relationship between NHHR and other basic clinical data, and collinearity analysis was performed to identify variables significantly associated with NHHR. Linear regression and correlation analyses were conducted to explore the relationships between NHHR, other lipid parameters, and GS. RCS with four knots was used to visualize the nonlinear relationship between continuous NHHR and high GS. Univariate and multivariate logistic regression analyses were used to identify risk factors for high GS. Three logistic regression models were constructed: Model I (unadjusted), Model II (adjusted for age, sex, and BMI), and Model III (further adjusted for variables with *P* < 0.05 in univariate analysis and non-collinearity [VIF < 5]). The independence of continuous and quartile NHHR in relation to high GS was analyzed. The Q1 was used as the reference group, and the OR and 95% confidence interval (CI) for the other quartiles were calculated. ROC curves were used to compare the predictive value of NHHR and other lipid parameters for high GS.

Based on logistic regression results and clinical experience, seven variables were selected to construct the clinical prediction model. Model 1 included NHHR + Model 2, and Model 2 included age, BNP, lipoprotein(a), albumin, glucose, and LVEF. The DeLong method was used to compare the area under the curve (AUC) of the model before and after incorporating NHHR. A nomogram was created to visualize the model. Internal validation was performed using the bootstrap method. The model’s predictive ability was assessed using the concordance index (C-index or AUC), calibration plots were used to evaluate predictive accuracy, and decision curve analysis (DCA) was used to assess clinical applicability. Finally, sensitivity analysis, including subgroup analysis and interaction analysis, was conducted.

The above statistical analyses were performed using SPSS Statistics (version 27) and R software (version 4.4.2). All statistical tests were two-sided, and *P* < 0.05 was considered statistically significant.

## Results

### Baseline characteristics

A total of 1799 patients were included in the study, with 907 cases in the high GS group and 892 cases in the low GS group. The comparison of patient characteristics between the two groups is shown in Table 1: the median age of patients was 71 (58–87) years, and 1312 (72.9%) were male. The high GS group had a higher proportion of males and older age. Compared with the low GS group, patients in the high GS group had higher levels of D-Dimer, BNP, creatinine, HbA1c, FBG, hs-CRP, red blood cell distribution width coefficient of variation (RBC-CV), platelet distribution width (PDW), LVEDD, Apolipoprotein B, Lipoprotein(a), LDL-C, TC, Apolipoprotein B/A1, and NHHR, and lower albumin and LVEF. Additionally, the high GS group had a higher incidence of previous coronary artery bypass grafting, type 2 diabetes, heart failure, cerebrovascular disease, and more frequent use of medications such as aspirin and ticagrelor.

**Table 1:** Baseline characteristics

Variables	Total (*n* = 1799)	High Gensini score (*n* = 709)	Low Gensini score (*n* = 892)	*P*	
**Demographic Characteristic**					
Age (year)	71 (58–87)	65 (57–71)	67 (58–73)	0.004	
Male, n (%)	1312 (72.9%)	685 (75.5%)	627 (70.3%)	0.013	
BMI (kg/m2)	25.06 (23.11–27.68)	25. 06 (23.05–27.64)	25.18 (23.11–27.68)	0.739	
Smoke history, n (%)	919 (51.1%)	472 (52.0%)	447 (50.1%)	0.423	
Alcohol history, n (%)	661 (36.7%)	338 (35.6%)	323 (37.9%)	0.328	
MAP (mmHg)	102 (92–112)	101 (91–112)	102 (93–112)	0.166	
**Comorbidities**,** n (%)**					
Old myocardial infarction	170 (9.4%)	95 (10.5%)	75 (8.4%)	0.147	
PCI history	324 (18.0%)	166 (18.3%)	158 (17.7%)	0.759	
CABG history	39 (2.2%)	34 (3.7%)	5 (0.6%)	< 0.001	
Hypertension	1132 (62.9%)	573 (63.2%)	559 (62.7%)	0.845	
Type 2 Diabetes	636 (35.4%)	354 (39.0%)	282 (31.6%)	0.001	
Atrial fibrillation	78 (4.3%)	44 (4.9%)	34 (3.8%)	0.299	
Heart failure	88 (4.9%)	61 (6.7%)	27 (3.0%)	< 0.001	
Cerebrovascular disease	311 (17.3%)	178 (19.6%)	133 (14.9)	0.009	
Hypohepatia	27 (1.5%)	18 (2.0%)	9 (1.0%)	0.120	
Renal insufficiency	96 (5.3%)	57 (6.3%)	39 (4.4%)	0.075	
**Laboratory Examination**					
ApolipoproteinA1 (mmol/L)	0.90 (0.80-1.00)	0.90 (0.80-1.00)	0.90 (0.80–1.10)	< 0.001	
Apolipoprotein B (mmol/L)	0.82 (0.67-1.00)	0.84 (0.69–1.04)	0.80 (0.65–0.97)	< 0.001	
Lipoprotein (a) (nmol/L)	155.10 (72.10-368.20)	175.20 (80.80-402.10)	145.20 (67.22–323.30)	< 0.001	
LDL-C (mmol/L)	2.56 (1.98–3.20)	2.63 (2.07–3.28)	2.49 (1.87–3.12)	< 0.001	
HDL-C (mmol/L)	0.94 (0.80–1.10)	0.93 (0.80–1.09)	0.95 (0.81–1.11)	0.082	
Triglyceride (mmol/L)	1.53 (1.15–2.16)	1.55 (1.16–2.16)	1.51 (1.14–2.16)	0.717	
Total cholesterol (mmol/L)	4.34 (3.64–5.13)	4.40 (3.55–5.07)	4.37 (3.76–5.17)	0.002	
Apolipoprotein B/A1	0.91 (0.71–1.15)	0.96 (0.76–1.20)	0.88 (0.66–1.09)	< 0.001	
TG/HDL	1.66 (1.12–2.48)	1.70 (1.16–2.53)	1.63 (1.08–2.44)	0.210	
NHHR	3.59 (2.75–4.51)	3.77 (2.90–4.70)	3.42 (2.61–4.33)	< 0.001	
D-Dimer (mg/L)	0.27 (0.15–0.63)	0.32 (0.16–0.80)	0.24 (0.13–0.51)	< 0.001	
BNP (pg/mL)	129 (40–398)	186 (56–628)	80 (33–238)	< 0.001	
Creatinine (umol/L)	71 (58–87)	72 (60–91)	68 (57–83)	< 0.001	
Uric Acid (umol/L)	341 (280–418)	342 (285–418)	337 (274–418)	0.150	
HbA1c (%)	6.20 (5.70–7.20)	6.30 (5.80–7.50)	6.10 (5.70–6.90)	0.001	
FBG (mmol/L)	5.90 (5.00-7.70)	6.30 (5.09–8.40)	5.70 (5.00–7.00)	< 0.001	
Albumin (g/L)	38 (35–41)	38 (35–41)	39 (36–42)	0.001	
hs-CRP (mg/L)	4.39 (1.50-12.61)	5.86 (1.93–17.22)	3.28 (1.27–8.86)	< 0.001	
Platelet (10^9/L)	218 (184–263)	218 (183–260)	218 (184–264)	0.664	
RBC-CV (10^12/L)	12.7 (12.3–13.2)	12.8 (12.3–13.3)	12.7 (12.3–13.2)	0.013	
PDW (10^9/L)	11.4 (10.2–12.9)	11.5 (10.3–12.9)	11.2 (10.1–12.8)	0.048	
LVEF (%)	52 (45–60)	48 (42–58)	58 (49–60)	< 0.001	
LVD (mm)	48 (45–52)	48 (45–53)	48 (45–51)	< 0.001	
**Intervene**,** n (%)**					
PCI	1296 (72.0%)	728 (80.3%)	568 (63.7%)	< 0.001	
PTCA	464 (25.8%)	274 (30.2%)	190 (21.3%)	< 0.001	
**Drugs**,** n (%)**					
Aspirin	1704 (94.7%)	882 (97.2%)	822 (92.2%)	< 0.001	
Copidogrel	919 (51.1%)	452 (49.8%)	467 (52.4%)	0.299	
Ticagrelor	767 (42.6%)	432 (47.6%)	335 (37.6%)	< 0.001	
Rosuvastatin	1370 (76.2%)	707 (77.9%)	663 (74.3%)	0.077	
Atorvastatin	334 (18.6%)	161 (17.8%)	173 (19.4%)	0.396	
PCSK9i	212 (11.8%)	113 (12.5%)	99 (11.1%)	0.381	
SGLT2i	257 (14.3%)	141 (15.5%)	116 (13.0%)	0.138	
GLP-1RA	51 (2.8%)	32 (3.5%)	19 (2.1%)	0.088	

Abbreviations: BMI, body mass index; MAP, mean arterial pressure; PCI, percutaneous coronary intervention; CABG, coronary artery bypass grafting; LDL-C, low-density lipoprotein cholesterol; HDL-C, high-density lipoprotein cholesterol; TG, triglyceride; NHHR, Non-High-Density Lipoprotein Cholesterol to High-Density Lipoprotein Cholesterol Ratio; BNP, brain natriuretic peptide; HbA1c, glycated hemoglobin A1c; FBG, fasting blood glucose; hs-CRP, high sensitivity C-reactive protein; RBC-CV, red blood cell distribution width coefficient of variation; PDW, platelet distribution width; LVEF, left ventricular ejection fraction; LVEDD, left ventricular end-diastolic dimension; PCSK9i, presenilin converting enzyme kexin-9 inhibitor; SGLT2i, sodium-dependent glucose transporters 2 inhibitors; GLP-1RA, glucagon-like peptide-1 receptor agonists

### Correlation analysis between NHHR and basic information

**Fig. 2:**
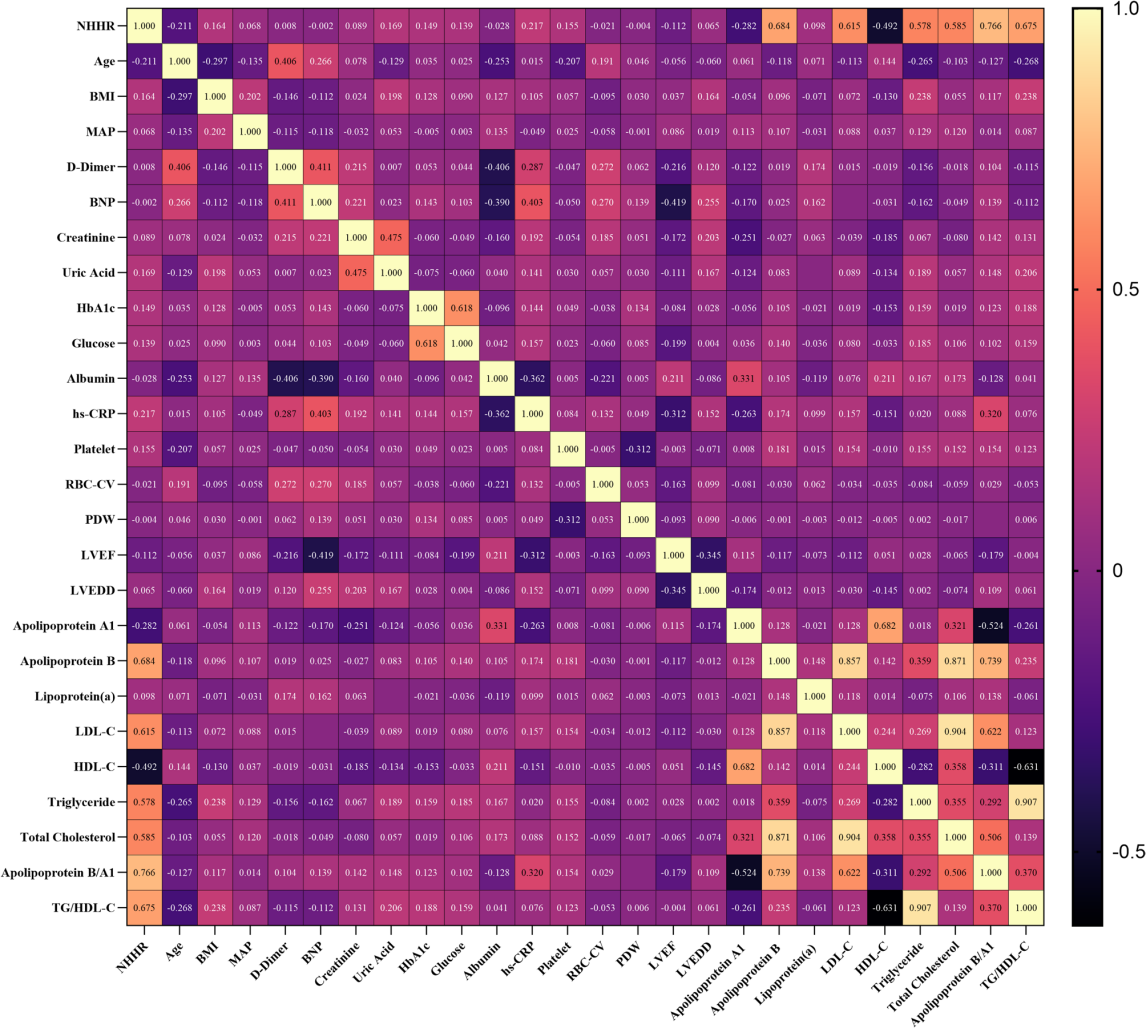
Spearman correlation test between NHHR and continuous variables. Abbreviations: BMI, body mass index; MAP, mean arterial pressure; LDL-C, low-density lipoprotein cholesterol; HDL-C, high-density lipoprotein cholesterol; TG, triglyceride; NHHR, Non-High-Density Lipoprotein Cholesterol to High-Density Lipoprotein Cholesterol Ratio; BNP, brain natriuretic peptide; hs-CRP, high sensitivity C-reactive protein; RBC-CV, red blood cell distribution width coefficient of variation; PDW, platelet distribution width; LVEF, left ventricular ejection fraction; LVEDD, left ventricular end-diastolic dimension

To explore the relationship between NHHR and baseline characteristics, spearman rank correlation analysis was conducted (Fig. 2). NHHR was positively correlated with BMI, mean arterial pressure, creatinine, uric acid, HbA1c, FBG, hs-CRP, Platelet, LVEDD, Apolipoprotein B, Lipoprotein (a), LDL-C, TG, TC, Apolipoprotein B/A1, and TG/HDL. It was negatively correlated with age, LVEF, Apolipoprotein A1, and HDL-C (Supplementary Table 2).

### Covariance analysis of NHHR with basic information

We conducted collinearity analysis on significant variables from the univariate analysis to determine whether strong correlations (VIF > 5) existed among them. NHHR exhibited high collinearity with Apolipoprotein B (tolerance: 0.108, VIF: 9.246), LDL-C (tolerance: 0.150, VIF: 6.670), TC (tolerance: 0.148, VIF: 6.765), and Apolipoprotein B/A1 (tolerance: 0.155, VIF: 6.437) (Supplementary Table 4).

### Logistic regression analysis of NHHR and high GS group

**Fig. 3:**
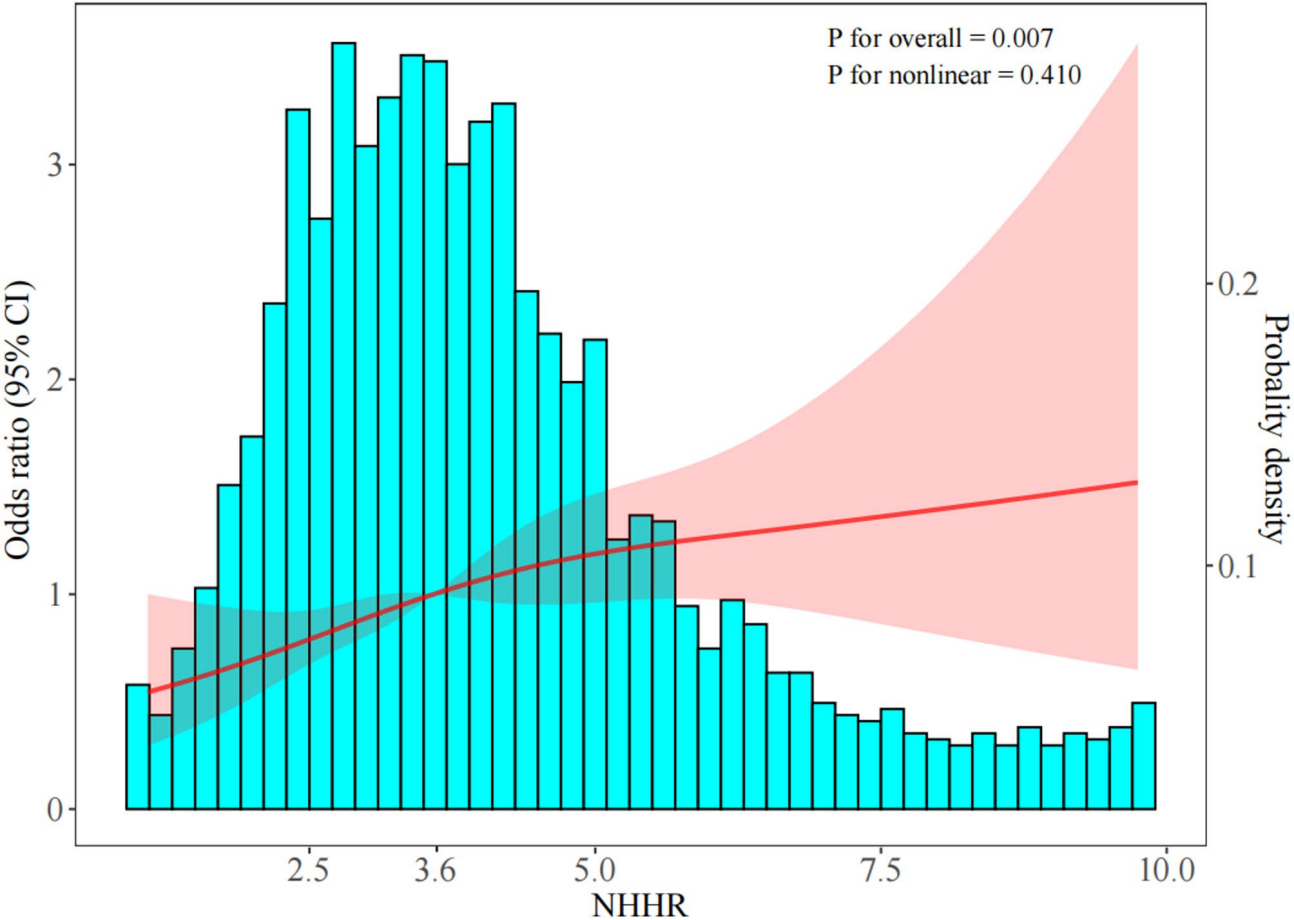
Restricted cubic spline analyses for NHHR and high GS. Abbreviations: NHHR, Non-High-Density Lipoprotein Cholesterol to High-Density Lipoprotein Cholesterol Ratio; GS, Gensini score

Restricted cubic spline diagram showed linear relationship between NHHR and high GS (Fig. 3). After univariate analysis, multivariate logistic regression analysis included variables with *P* < 0.05 from the univariate analysis and BMI, while excluding highly collinear variables such as Apolipoprotein B, LDL-C, TC, and Apolipoprotein B/A1 (VIF > 5). The results indicated that NHHR (OR = 1.145, CI: 1.055–1.243, *P* = 0.001) was an independent risk factor (Supplementary Table 3).

Based on NHHR quartiles, the patients were divided into four groups: Q1: NHHR < 2.75, Q2: 2.75 ≤ NHHR < 3.59, Q3: 3.59 ≤ NHHR < 4.51, Q4: NHHR ≥ 4.51. The results of Model III showed that after adjusting for the above confounding factors, the risk of severe coronary stenosis (high GS) in ACS patients increased with higher NHHR levels. Using Q1 as the reference group, Q2: OR = 1.16 (95% CI: 0.86–1.56, *P* = 0.331); Q3: OR = 1.36 (95% CI: 1.01–1.84, *P* = 0.044); Q4: OR = 1.66 (95% CI: 1.21–2.28, *P* = 0.002) (Table 2).

**Table 2:** Logistic regression of NHHR and its quartiles with high GS

Variables	Model I			Model II				Model III			
	OR (95% CI)	*P*		OR (95% CI)		*P*		OR (95% CI)		*P*	
Continuous NHHR	1.19 (1.11–1.27)		< 0.001		1.23 (1.15–1.33)		< 0.001		1.15 (1.06–1.25)		0.001
Quartiles of NHHR			< 0.001				< 0.001				0.013
Q1	*Reference*				*Reference*				*Reference*		
Q2	1.27 (0.98–1.66)		0.071		1.31 (1.01–1.70)		0.049		1.16 (0.86–1.56)		0.331
Q3	1.48 (1.14–1.92)		0.004		1.56 (1.19–2.03)		0.001		1.36 (1.01–1.84)		0.044
Q4	1.92 (1.48–2.50)		< 0.001		2.18 (1.66–2.87)		< 0.001		1.66 (1.21–2.28)		0.002

Model I: Unadjusted;

Model II: Based on Model I further adjusted for age, male, body mass index;

Model III: Based on Model II further adjusted for age, male, body mass index, coronary artery bypass grafting, type 2 diabetes, heart failure, cerebrovascular disease, percutaneous coronary intervention, percutaneous transluminal coronary angioplasty, aspirin, tegretol, D-Dimer, brain natriuretic peptide, creatinine, glycated hemoglobin A1c, fasting blood glucose, albumin, high sensitivity C-reactive protein, left ventricular ejection fraction, left ventricular end-diastolic diameter, Lipoprotein (a), Apolipoprotein A1. Abbreviations: NHHR, Non-High-Density Lipoprotein Cholesterol to High-Density Lipoprotein Cholesterol Ratio; OR, odds ratio; GS, Gensini score

### Correlation analysis between GS and lipid parameters

Univariate linear regression and Spearman rank correlation analysis (Supplementary Fig. 1) showed: The GS was positively correlated with NHHR (*r* = 0.10), TC (*r* = 0.07), LDL-C (*r* = 0.10), Lipoprotein (a) (*r* = 0.14), Apolipoprotein B (*r* = 0.08), and Apolipoprotein B/A1 (*r* = 0.12) (*P* < 0.05), and negatively correlated with Apolipoprotein A1 (*r*= −0.10) (*P* < 0.05).

### Predictive value of different lipid parameters for high GS

**Fig. 4:**
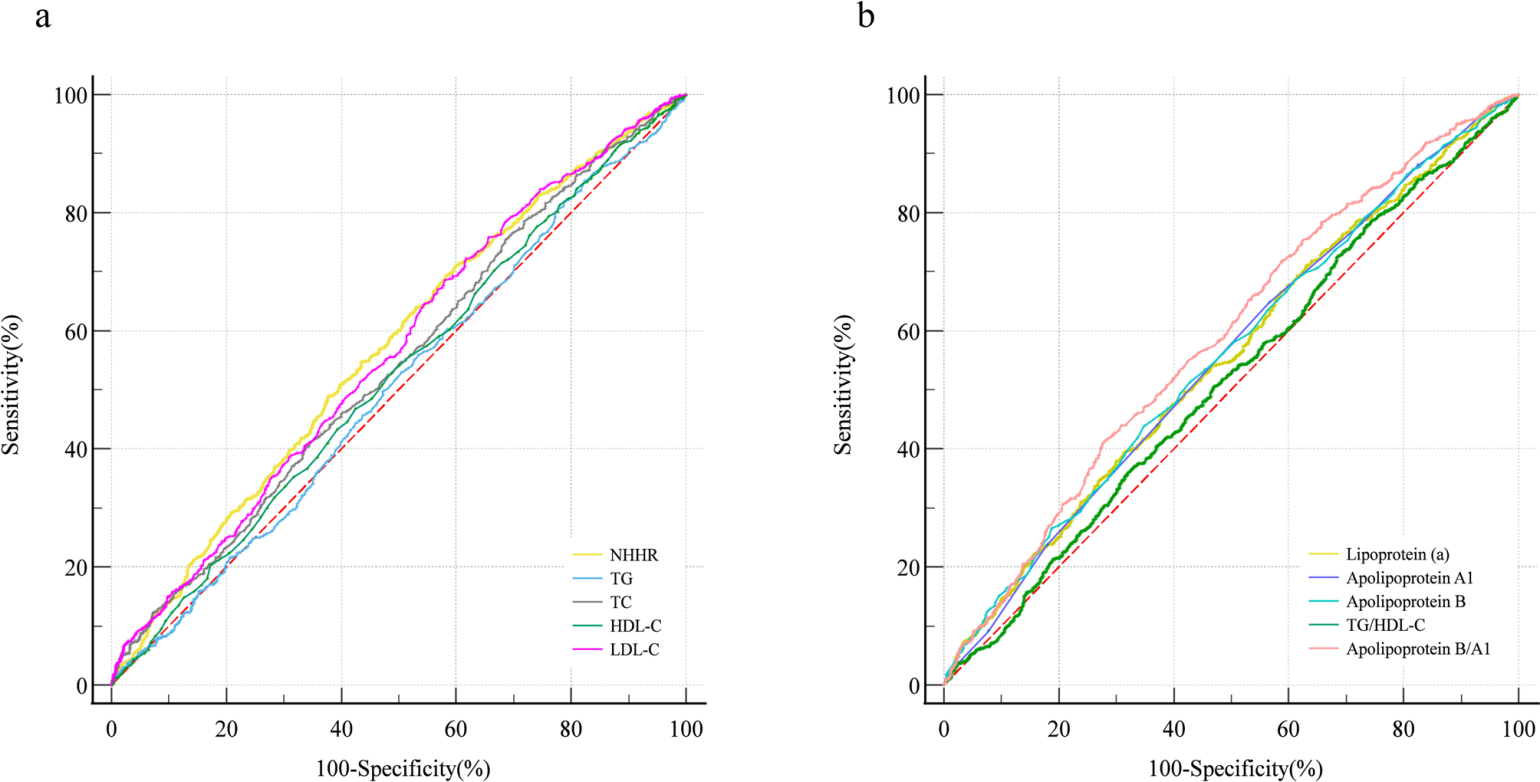
The ROC curve of different lipid parameters for predicting high GS (**a**,** b**). The ROC curve of Model 1 (with NHHR) and Mode 2 (without NHHR) for predicting high GS (**c**). Abbreviations: NHHR, Non-High-Density Lipoprotein Cholesterol to High-Density Lipoprotein Cholesterol Ratio; LDL-C, low-density lipoprotein cholesterol; HDL-C, high-density lipoprotein cholesterol; TC, triglyceride; TG, triglyceride; GS, Gensini score

The ROC curve analysis demonstrated the predictive value of NHHR and other lipid parameters for high GS (Fig. 4). NHHR alone predicted the presence of high GS in ACS patients with an AUC = 0.570 (95% CI: 0.547–0.593, *P* < 0.001), which was superior to traditional lipid parameters such as TC (AUC = 0.542, 95% CI: 0.519–0.565, *P* = 0.002), LDL-C (AUC = 0.563, 95% CI: 0.540–0.586, *P* < 0.001), Lipoprotein(a) (AUC = 0.552, 95% CI: 0.529–0.575, *P* < 0.001), TC (AUC = 0.542, 95% CI: 0.519–0.565, *P* = 0.002), Apolipoprotein B (AUC = 0.556, 95% CI: 0.533–0.579, *P* < 0.001), and ApolipoproteinA1 (AUC = 0.554, 95% CI: 0.530–0.577, *P* < 0.001). However, the AUC value of Apolipoprotein B/A1 was higher than that of NHHR, AUC = 0.586 (95% CI: 0.563–0.609, *P* < 0.001) (Table 3).

**Table 3:** Predictive efficacy of NHHR and different lipid parameters for high GS

Variables	AUC (95% Cl)	Sensitivity	Specificity	*P*
NHHR	0.570 (0.547–0.593)	54.69	56.50	< 0.001
Triglyceride (mmol/L)	0.505 (0.482–0.528)	85.45	17.60	0.717
Total cholesterol (mmol/L)	0.542 (0.519–0.565)	75.41	31.39	0.002
HDL-C	0.524 (0.500-0.547)	46.86	57.51	0.081
LDL-C	0.563 (0.540–0.586)	68.58	41.93	< 0.001
TG/HDL	0.517 (0.494–0.540)	77.95	26.46	0.210
Lipoprotein (a) (nmol/L)	0.552 (0.529–0.575)	39.36	68.72	< 0.001
ApolipoproteinB (mmol/L)	0.556 (0.533–0.579)	43.88	65.13	< 0.001
ApolipoproteinA1 (mmol/L)	0.554 (0.530–0.577)	64.83	43.39	< 0.001
Apolipoprotein B/A1	0.586 (0.563–0.609)	41.01	72.31	< 0.001

Abbreviations: NHHR, Non-High-Density Lipoprotein Cholesterol to High-Density Lipoprotein Cholesterol Ratio; LDL-C, low-density lipoprotein cholesterol; HDL-C, high-density lipoprotein cholesterol; TG, triglyceride; GS, Gensini score

### Nomogram of high GS in patients with ACS

**Fig. 5:**
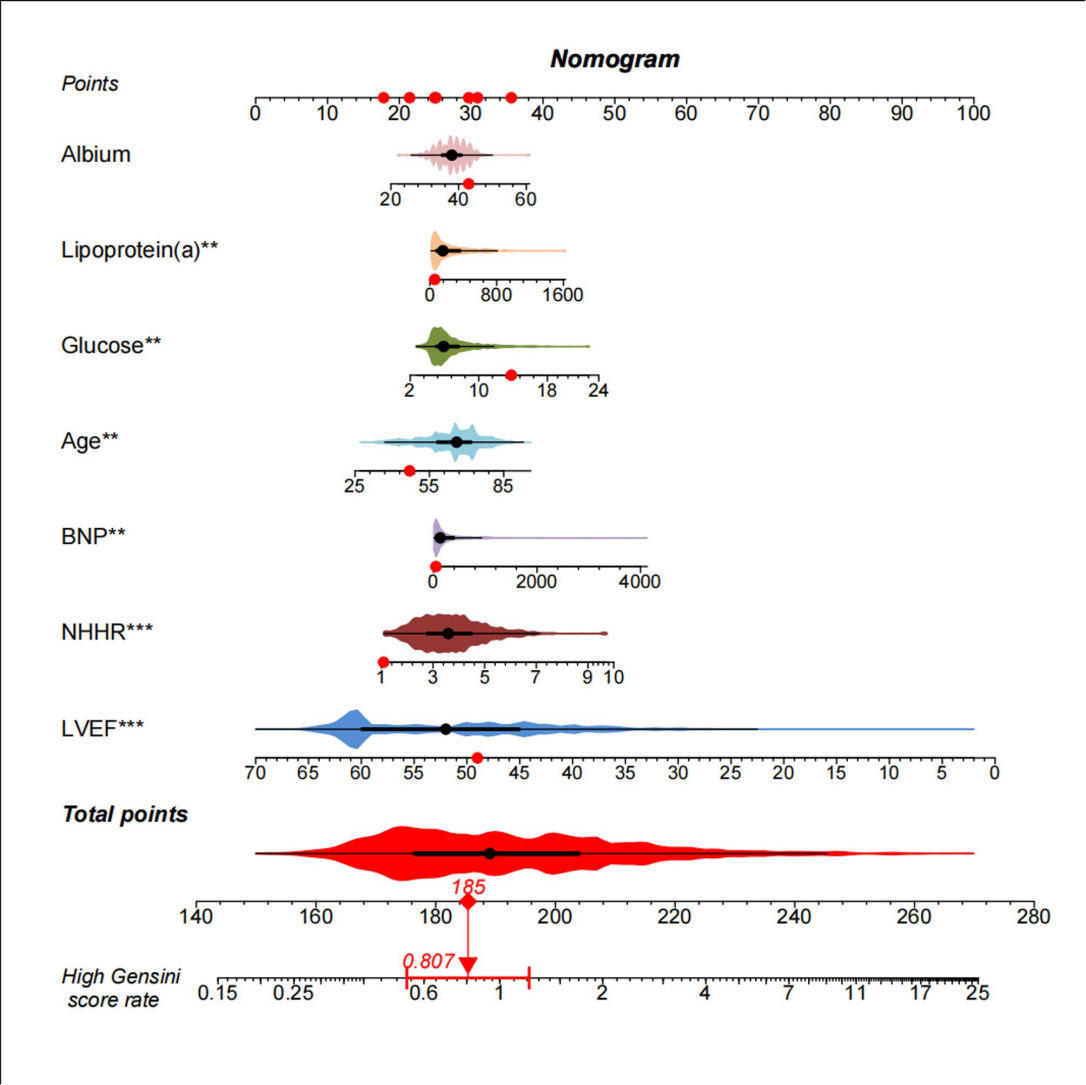
The Nomogram of Model 1 for predicting high GS. Abbreviations: NHHR, Non-High-Density Lipoprotein Cholesterol to High-Density Lipoprotein Cholesterol Ratio; BNP, brain natriuretic peptide, LVEF, left ventricular ejection fraction; GS, Gensini score

Based on the results of the multivariate logistic regression model, we selected seven risk factor variables to construct the high GS risk assessment model. Model 1 included NHHR, age, BNP, albumin, lipoprotein (a), glucose, and LVEF. A nomogram was developed to visualize Model 1, illustrating the contribution of each variable to predicting high GS group outcomes (Fig. 5). The higher the total points derived from the distribution of each variable in the nomogram, the greater the likelihood of severe coronary artery lesions in ACS patients.

### Evaluation and validation of the Nomogram

The ROC curve analysis for Model 1 showed an AUC of 0.703 (95% CI: 0.682–0.724). In Model 2, after removing NHHR, the AUC decreased to 0.696 (95% CI: 0.674–0.717). DeLong’s test indicated a statistically significant difference between Model 1 (with NHHR) and Model 2 (without NHHR) (Z = 2.144, *P* = 0.032). The C-index for Model 1 was calculated as 0.703, indicating that incorporating NHHR improved the predictive performance of the risk model, effectively distinguishing patients with varying severity of coronary stenosis (Supplementary Fig. 2a).

The accuracy and potential overfitting of the model were assessed using the bootstrap method with 1,000 resamples. The calibration curve showed that the predicted probability of severe coronary lesions in ACS patients closely aligned with the ideal diagonal line, indicating strong consistency between predicted and observed values and good model calibration (Supplementary Fig. 2b). Decision curve analysis (DCA) was used to evaluate the clinical applicability of the model, demonstrating that Model 1 has excellent clinical utility (Supplementary Fig. 2c).

#### Sensitivity analysis

Subgroup analysis was conducted based on age, sex, BMI, history of coronary artery bypass grafting or stent implantation, Lp(a), and ACS type to further investigate the association between NHHR and severe coronary lesions (Supplementary Table 5). Younger patients (< 65 years), males, those with high BMI (≥ 28 kg/m²), no history of coronary artery bypass grafting or stent implantation, elevated Lp(a) (≥ 125 nmol/L), and myocardial infarction (STEMI/UNSTEMI) showed a stronger risk association in the high-risk group (Q4). No statistically significant interaction effects were observed across subgroups (P for interaction > 0.05).

## Discussion

Our study was the first to explore NHHR as a risk predictor for high GS in ACS patients. The results showed that ACS patients with high GS had significantly higher NHHR values compared to those with low GS. After adjusting for various confounding factors, NHHR was found to be an independent risk factor for the high GS group. Moreover, as the NHHR value increased, the risk coefficient also rose. We also compared the predictive value of NHHR with that of conventional lipid parameters for high GS. Importantly, we found that NHHR, as a novel lipid metabolism index, outperformed traditional lipid risk factors. NHHR had an incremental effect in improving the predictive value of the model. Clinical prediction models incorporating NHHR showed good clinical utility in predicting the presence of more severe coronary lesions in patients with ACS.

The pathophysiological process of ACS primarily involves the formation, rupture or erosion of atherosclerotic plaques, thrombosis, and acute coronary artery occlusion, which is closely related to dyslipidemia [2]. Some modifiable risk factors, such as lipid disorders, have not been well controlled and are the main reasons for the persistently high incidence and mortality rates of ACS. A high GS indicates that patients may have more extensive multi-vessel disease and more severe stenosis, serving as an independent predictor of long-term cardiovascular events (such as recurrent myocardial infarction and cardiogenic death) in ACS patients [22]. Patients with higher GSs often require more aggressive interventional treatments (such as stent implantation or coronary artery bypass surgery), while those with lower scores can rely more on pharmacological treatment [23].

Multiple studies have shown that different lipid markers (such as LDL-C, HDL-C, ApoB, non-HDL-C, TG, etc.) are significantly associated with the GS, aiding in the risk assessment and prognostic management of coronary artery disease in ACS patients. For a long time, clinicians have regarded LDL-C as the primary intervention target for ASCVD patients. However, due to the time-consuming and costly nature of LDL-C testing, its susceptibility to TG levels, and the presence of residual cardiovascular risk even at low LDL-C levels it has limitations [24]. ApoB can also serve as an important target for the prevention and treatment of ASCVD, particularly in patients with mild to moderate hypertriglyceridemia (2–5.6 mmol/L), diabetes, obesity, or metabolic syndrome, or in patients with LDL-C levels < 1.8 mmol/L [25]. The unique structure and function of Apo(a) in Lp(a) make it more atherogenic than LDL, and it can serve as an independent risk factor for ASCVD [26, 27]. Research by Zahra et al. indicates that for every 1 mg/dL increase in apolipoprotein A-I and apolipoprotein B100 levels, the risk of a high Gensini score decreases by 7% and increases by 8%, respectively (*P* = 0.001) [28]. A study from China involving 826 patients with coronary heart disease showed that ApoB/ApoA1 was still significantly correlated with the adjusted Gensini score [29]. RELACS research indicates that elevated Lp(a) levels are associated with earlier onset and greater complexity of CAD in ACS patients, i.e., for every increase of one tertile in Lp(a), the Gensini score increases by 8.01 points (*P* = 0.019) [5]. In the recent years, researchers have found that LDL-C is not a perfect risk marker for ASCVD. Therefore, to further improve patient prognosis, attention has shifted to Non-HDL-C, which is less affected by TG variability and serves as a strong alternative marker for remnant cholesterol and a critical target for residual risk [30]. As increasing evidence from comparative studies of LDL-C and Non-HDL-C emerges, the latest domestic and international guidelines have begun to mention Non-HDL-C. The 2021 ESC guidelines recommend Non-HDL-C as a reasonable alternative treatment target for all patients, particularly those with hypertriglyceridemia or diabetes [31]. The 2021 CCS guidelines emphasize that Non-HDL-C can replace LDL-C as the preferred lipid parameter for screening patients with triglyceride levels > 1.5 mmol/L [32]. Although other lipid parameters have demonstrated prognostic value in patients with cardiovascular disease, they require specialized testing and may not be widely available. In contrast, NHHR can be easily obtained from routine lipid testing, making it a more accessible and cost-effective alternative, particularly in primary care or acute settings. NHHR not only considers Non-HDL-C but also incorporates HDL-C, providing a more comprehensive reflection of an individual’s cardiovascular risk. The study by Yu et al. found a U-shaped relationship between NHHR and all-cause mortality in U.S. adults with diabetes or prediabetes. When baseline NHHR exceeded the threshold (2.72 and 2.83), NHHR was positively correlated with all-cause mortality (hazard ratio, HR: 1.11, 95% CI: 1.06–1.16) and cardiovascular mortality (HR: 1.08, 95% CI: 1.00-1.16) [33]. A study of 2,253 CAD patients who underwent PCI showed a U-shaped relationship between NHHR and the incidence of MACCE, with the threshold for NHHR being 3.119 [34]. Our study confirmed and supplemented the finding that NHHR more accurately reflects the overall residual lipid status in ACS patients, emphasizing the importance of comprehensive lipid management and lipid-lowering therapy for patient prognosis. Given its simplicity and availability, NHHR could serve as a valuable adjunct to existing lipid management strategies, especially in settings where apolipoprotein assays or lipoprotein(a) testing are not routinely performed. Its integration into risk assessment models may enhance early identification of high-risk individuals, informing the intensity of lipid-lowering interventions. Similarly, our study demonstrated that NHHR had a predictive AUC of 0.570 for coronary artery stenosis, outperforming other lipid parameters such as LDL-C (0.563), lipoprotein(a) (0.552), Apo B (0.556), and TC (0.542).

Our study included 1,799 ACS patients for a comprehensive evaluation. Although the association between dyslipidemia and coronary artery disease is well established, our study provides additional clinical value by focusing specifically on the NHHR in patients with ACS. Unlike previous studies that have examined non-HDL-C and HDL-C as separate risk factors in general or stable CAD populations [35–37], our research highlights NHHR as a simple, integrated marker that correlates with angiographically confirmed lesion severity in an acute setting. This targeted approach not only reinforces current guideline-endorsed lipid parameters but also offers practical implications for risk stratification in ACS patients, where timely and accessible indicators are critical. Therefore, while our findings may be confirmatory in nature, they extend existing knowledge by validating NHHR as a clinically useful marker in a high-risk, under-investigated subgroup. This study constructed a nomogram model (Model 1) to predict severe coronary artery lesions using multivariate logistic regression analysis, selecting seven risk factor variables: NHHR, age, BNP, albumin, lipoprotein(a), glucose, and LVEF. The study found that after incorporating NHHR, the AUC of Model 1 was significantly higher than that of Model 2, which excluded NHHR (0.703 vs. 0.696, *P* = 0.032). The calibration curve, evaluated using the bootstrap method, showed good agreement between predicted and observed values, further confirming the stability and reliability of the model. Moreover, decision curve analysis indicated that Model 1 had good net benefit across a wide range of threshold probabilities, demonstrating its high clinical utility. In summary, the risk assessment model incorporating NHHR effectively improves the prediction of coronary lesion severity in ACS patients, providing a scientific basis for early clinical identification and intervention. However, the model requires further validation in larger sample sizes and external datasets to confirm its generalizability and robustness. Subgroup analysis revealed that NHHR exhibited stronger predictive power in certain populations, including younger patients (< 65 years), males, those with high BMI (≥ 28 kg/m²), no history of bypass or stent implantation, elevated Lp(a) (≥ 125 nmol/L), and myocardial infarction (STEMI/UNSTEMI) patients. This suggested that NHHR may have greater predictive value in these high-risk populations. Lp(a) may exacerbate plaque instability through pro-inflammatory, pro-thrombotic mechanisms and oxidative modification of LDL. It may act synergistically with NHHR to amplify plaque burden [38]. Individuals with genetically elevated Lp(a) levels may be more sensitive to changes in lipid ratios [39].

However, our study had some limitations. First, this was a single-center, retrospective study, which may not be fully generalizable to other regions or populations. Future large-scale, multicenter, prospective studies may be needed to validate our findings. Second, although we adjusted for various confounding factors, our study may still be subject to selection bias and information bias, and it was not possible to fully control for all potential confounders, such as lifestyle and family history, which could affect the interpretation of the results. Third, although the GS is widely used, the scoring system still relies on imaging assessments, which may involve operational differences or subjective variability between evaluators. Fourth, since our study lacked some conventional inflammatory markers and detailed lipid subfractions, prospective studies incorporating a broader range of inflammatory and lipid biomarkers in the future will help to validate and expand our findings. Finally, while we developed a nomogram model, its utility remains uncertain due to the absence of external validation. Future studies should focus on prospectively evaluating the model’s performance through external data collection.

## Conclusion

This study found that higher NHHR were associated with more severe coronary artery stenosis in ACS patients (Gensini score > 68.5), and NHHR remained an independent risk factor after adjusting for confounding factors. The risk of severe coronary stenosis significantly increased with rising NHHR. NHHR showed superior predictive performance for the severity of coronary lesions. Incorporating NHHR into the predictive model improved its efficiency and clinical applicability. While our findings suggest that NHHR may serve as a useful marker for assessing the severity of coronary artery disease in ACS patients, further large-scale, multi-center studies are warranted to validate its predictive value across diverse populations and clinical settings.

## Electronic supplementary material

**Supplementary Material 1:** Supplementary Figure 1. Correlation between NHHR and different blood lipids.
Supplementary Figure 2. The ROC curve of model 1 and model 2 for predicting high Gensini score (a) and calibration plots (b) and decision curve analysis (c) of Model 1
Supplementary Table 1. Gensini score rules
Supplementary Table 2. Correlation between NHHR and continuous variables
Supplementary Table 3. Univariate and multivariate analyses of ACS patients with high Gensini score
Supplementary Table 4. Collinearity analysis of risk factors with NHHR
Supplementary Table 5. Subgroups analyze

## Data Availability

No datasets were generated or analysed during the current study.

## Abbreviations

NHHR: Non-high-density lipoprotein cholesterol to high-density lipoprotein cholesterol ratio
ACS: Acute coronary syndrome
GS: Gensini score
ROC: Receiver operating characteristic
STEMI: ST-elevation myocardial infarction
NSTEMI: Non-ST-elevation myocardial infarction
UA: Unstable angina
LDL-C: Low-density lipoprotein cholesterol
HDL-C: High-density lipoprotein cholesterol
CAG: Angiography
MI: Myocardial infarction
LVEF: Left ventricular ejection fraction
LVEDD: Left ventricular end-diastolic diameter
PCSK9i: Proprotein convertase subtilisin/kexin type 9 inhibitors
SGLT2i: Sodium-glucose cotransporter 2 inhibitors
GLP-1RA: GLP-1 receptor agonists
ApoA1: Apolipoprotein
ApoB: Apolipoprotein B
Lp(a): Lipoprotein (a)
TG: Triglycerides
TC: Total cholesterol
BNP: Brain natriuretic peptide
FBG: Fasting blood glucose
HbA1c: Glycated hemoglobin
hs-CRP: Hypersensitive C-reactive protein
BMI: Body mass index
MAP: Mean arterial pressure
CI: Confidence interval
OR: Odds ratio
DCA: Decision curve analysis
C-index: Concordance index
CCU: Coronary care unit
RBC-CV: Red blood cell distribution width coefficient of variation
PDW: Platelet distribution width
HR: Hazard ratio
