# Supplementary material for: Exploring the relationship between NHHR and the degree of coronary artery stenosis in patients with acute coronary syndromes
Source: BMC Cardiovasc Disord. 2025 Aug 7;25:589. doi: 10.1186/s12872-025-05066-z (PMC12330120; doi:10.1186/s12872-025-05066-z)
Supplement: Supplementary file 1 — Supplementary Material 1: Supplementary Figure 1. Correlation between NHHR and different blood lipids. Supplementary Figure 2. The ROC curve of model 1 and model 2 for predicting high Gensini score (a) and calibration plots (b) and decision curve analysis (c) of Model 1 Supplementary Table 1. Gensini score rules Supplementary Table 2. Correlation between NHHR and continuous variables Supplementary Table 3. Univariate and multivariate analyses of ACS patients with high Gensini score Supplementary Table 4. Collinearity analysis of risk factors with NHHR Supplementary Table 5. Subgroups analyze [file 12872_2025_5066_MOESM1_ESM.docx]

**Supplementary Table 1. Gensini score rules**

| **Lesion site** | | **Ratio** | |  | | **Narrowing** | **Score** |
| --- | --- | --- | --- | --- | --- | --- | --- |
| LM |  | 5 |  | |  | ≤ 25％ 1 | 1 |
| LAD | Proximal | 2.5 | D1 | | 1 | 26％～50％ | 2 |
|  | Middle | 2.5 | D2 | | 0.5 | 51％～75％ | 4 |
|  | Distal | 1 | D3 | | 0.5 | 76％～90％ | 8 |
| LCX | Proximal | 2.5 | OM | | 1 | 91%～99% | 16 |
|  | Middle | 1 | PL | | 0.5 | 100% | 32 |
|  | Distal | 1 |  | |  |  |  |
| RCA | Proximal | 1 | PDA | | 1 |  |  |
|  | Middle | 1 | PL | | 0.5 |  |  |
|  | Distal | 1 |  | |  |  |  |

Abbreviations: LM: left main coronary artery; LAD: left anterior descending branch; LCX: left circumflex branch; RCA: right coronary artery; D: diagonal branches; OM: obtuse marginal branch; PL: left posterior ventricular branches; PDA: posterior descending artery.

**Supplementary Table 2. Correlation between NHHR and continuous variables**

| **Variables** | **Correlation coefficient** | |
| --- | --- | --- |
|  | ***r*** | ***P*** |
| Age (year) | -0.211 | < 0.001* |
| BMI (kg/m2) | 0.164 | < 0.001* |
| MAP (mmHg) | 0.068 | 0.004* |
| D-Dimer (mg/L) | 0.008 | 0.737 |
| BNP (pg/mL) | -0.002 | 0.940 |
| Creatinine (umol/L) | 0.089 | < 0.001* |
| Uric Acid (umol/L) | 0.169 | < 0.001* |
| HbA1c (%) | 0.149 | < 0.001* |
| FBG (mmol/L) | 0.139 | < 0.001* |
| Albumin (g/L) | -0.028 | 0.240 |
| hs-CRP (mg/L) | 0.217 | < 0.001* |
| Platelet (10^9/L) | 0.155 | < 0.001* |
| RBC-CV (10^12/L) | -0.021 | 0.362 |
| PDW (10^9/L) | -0.004 | 0.852 |
| LVEF (%) | -0.112 | < 0.001* |
| LVEDD (mm) | 0.065 | 0.006* |
| ApolipoproteinA1 (mmol/L) | -0.282 | < 0.001* |
| ApolipoproteinB (mmol/L) | 0.684 | < 0.001* |
| Lipoprotein (a) (mmol/L) | 0.098 | < 0.001* |
| LDL-C (mmol/L) | 0.615 | < 0.001* |
| HDL-C (mmol/L) | -0.492 | < 0.001* |
| Triglyceride (mmol/L) | 0.578 | < 0.001* |
| Total cholesterol (mmol/L) | 0.585 | < 0.001* |
| Apolipoprotein B/A1 | 0.766 | < 0.001* |
| TG/HDL | 0.675 | < 0.001* |

Abbreviations: BMI, body mass index; MAP, mean arterial pressure; LDL-C, low-density lipoprotein cholesterol; HDL-C, high-density lipoprotein cholesterol; TG, triglyceride; BNP, brain natriuretic peptide; HbA1c, glycated hemoglobin A1c; FBG, fasting blood glucose; hs-CRP, high sensitivity C-reactive protein; RBC-CV, red blood cell distribution width coefficient of variation; PDW, platelet distribution width; LVEF, left ventricular ejection fraction; LVEDD, left ventricular end-diastolic dimension. *<0.05

**Supplementary Table 3. Univariate and multivariate analyses of ACS patients with high Gensini score**

| **Variables** | **Univariate analysis** | | | **Multivariate analysis** | | |
| --- | --- | --- | --- | --- | --- | --- |
|  | ***OR (95% CI)*** | ***P*** | ***B*** | ***OR (95% CI)*** | ***P*** | ***B*** |
| **Demographic Characteristic** | | | | | | |
| Age (year) | 1.012 (1.004-1.020) | 0.004* | 0.012 | 1.020 (1.010-1.031) | < 0.001* | 0.020 |
| Male, n (%) | 1.304 (1.059-1.607) | 0.013* | 0.266 | 1.192 (0.919-1.547) | 0.186 | 0.176 |
| BMI (kg/m2) | 1.003 (0.981-1.025) | 0.778 | 0.003 | 1.007 (0.980-1.034) | 0.628 | 0.007 |
| Smoke history, n (%) | 1.080 (0.898-1.300) | 0.414 | 0.077 | - | - | - |
| Alcohol history, n (%) | 0.907 (0.748-1.098) | 0.316 | -0.098 | - | - | - |
| MAP (mmHg) | 0.997(0.991-1.003) | 0.306 | -0.003 | - | - | - |
| **Comorbidities, n (%)** |  |  |  |  |  |  |
| Old myocardial infarction | 1.274 (0.927-1.751) | 0.135 | 0.243 | - | - | - |
| PCI history | 1.041 (0.818-1.324) | 0.745 | 0.040 | - | - | - |
| CABG history | 6.909 (2.690-17.747) | < 0.001* | 1.933 | 6.189 (2.280-16.800) | < 0.001* | 1.823 |
| Hypertension | 1.022 (0.844-1.237) | 0.824 | 0.022 | - | - | - |
| Type 2 Diabetes | 1.385 (1.140-1.682) | 0.001* | 0.325 | 1.225 (0.929-1.616) | 0.151 | 0.203 |
| Atrial fibrillation | 1.287 (0.814-2.033) | 0.280 | 0.252 | - | - | - |
| Heart failure | 2.310 (1.454-3.670) | < 0.001* | 0.837 | 1.322 (0.998-1.459) | 0.525 | -0.180 |
| Cerebrovascular disease | 1.393 (1.089-1.783) | 0.008* | 0.332 | 1.302 (0.998-1.722) | 0.064 | 0.279 |
| Hypohepatia | 1.987 (0.888-4.446) | 0.095 | 0.686 | - | - | - |
| Renal insufficiency | 1.467 (0.965-2.228) | 0.073 | 0.383 | - | - | - |
| **Intervene, n (%)** |  |  |  |  |  |  |
| PCI | 2.320 (1.875-2.871) | < 0.001* | 0.842 | 2.291 (1.765-2.975) | < 0.001* | 0.829 |
| PTCA | 1.590 (1.292-1.955) | < 0.001* | 0.463 | 2.055 (1.596-2.647) | < 0.001* | 0.720 |
| **Drugs, n (%)** |  |  |  |  |  |  |
| Aspirin | 3.004 (1.8841-4.790) | < 0.001* | 1.100 | 1.728 (0.990-3.107) | 0.054 | 0.547 |
| Copidogrel | 0.904 (0.751-1.088) | 0.285 | -0.101 | - | - | - |
| Ticagrelor | 1.512 (1.253-1.825) | < 0.001* | 0.414 | 1.429 (1.135-1.800) | 0.002* | 0.357 |
| Rosuvastatin | 1.221 (0.983-1.517) | 0.072 | 0.200 | - | - | - |
| Atorvastatin | 0.897 (0.707-1.138) | 0.370 | -0.109 | - | - | - |
| PCSK9i | 1.140 (0.855-1.519) | 0.371 | 0.131 | - | - | - |
| SGLT2i | 1.231 (0.945-1.605) | 0.124 | 0.208 | - | - | - |
| GLP-1RA | 1.680 (0.945-2.987) | 0.077 | 0.519 | - | - | - |
| **Laboratory Examination** |  |  |  |  |  |  |
| D-Dimer (mg/L) | 1.081 (1.023-1.141) | 0.005* | 0.078 | 0.972 (0.913-1.035) | 0.380 | -0.028 |
| BNP (pg/mL) | 1.001 (1.001-1.001) | < 0.001* | 0.001 | 1.000 (1.000-1.001) | 0.005* | 0.001 |
| Creatinine (umol/L) | 1.001 (1.000-1.002) | 0.023* | 0.001 | 0.999 (0.998-1.001) | 0.251 | -0.001 |
| Uric Acid (umol/L) | 1.001 (1.000-1.001) | 0.150 | 0.001 | - | - | - |
| HbA1c (%) | 1.120 (1.047-1.198) | 0.001* | 0.113 | 0.907 (0.807-1.020) | 0.105 | -0.097 |
| FBG (mmol/L) | 1.103 (1.067-1.140) | < 0.001* | 0.098 | 1.059(1.007-1.114) | 0.026* | 0.058 |
| Albumin (g/L) | 0.977 (0.959-0.995) | 0.012* | -0.024 | 1.026 (1.001-1.052) | 0.038* | 0.026 |
| hs-CRP (mg/L) | 1.0012 (1.008-1.017) | < 0.001* | 0.012 | 1.005 (1.000-1.010) | 0.061 | 0.005 |
| Platelet (10^9/L) | 1.000 (0.998-1.001) | 0.609 | 0.000 | - | - | - |
| RBC-CV (10^12/L) | 1.075 (0.985-1.173) | 0.104 | 0.072 | - | - | - |
| PDW (10^9/L) | 1.036 (0.991-1.083) | 0.116 | 0.036 | - | - | - |
| LVEF (%) | 0.933 (0.923-0.943) | < 0.001* | -0.070 | 0.950 (1.000-1.010) | < 0.001* | -0.051 |
| LVEDD (mm) | 1.042 (1.026-1.058) | < 0.001* | 0.041 | 1.007 (0.991-1.024) | 0.421 | 0.007 |
| **Lipid parameters** |  |  |  |  |  |  |
| ApolipoproteinA1 (mmol/L) | 0.423 (0.283-0.633) | < 0.001* | -0.860 | 0.741 (0.446-1.231) | 0.247 | -0.300 |
| Apolipoprotein B (mmol/L) | 2.314 (1.605-3.335) | < 0.001* | 0.839 | - | - | - |
| Lipoprotein (a) (mmol/L) | 1.001 (1.000-1.001) | < 0.001* | 0.001 | 1.000 (1.000-1.001) | 0.031* | 0.001 |
| LDL-C (mmol/L) | 1.297 (1.173-1.434) | < 0.001* | 0.260 | - | - | - |
| HDL-C (mmol/L) | 1.001 (0,998-1.004) | 0.555 | 0.001 | - | - | - |
| Triglyceride (mmol/L) | 1.020 (0.943-1.103) | 0.627 | 0.019 | - | - | - |
| Total cholesterol (mmol/L) | 1.162 (1.075-1.257) | < 0.001* | 0.151 | - | - | - |
| Apolipoprotein B/A1 | 2.127 (1,630-2.775) | < 0.001* | 0.755 | - | - | - |
| TG/HDL | 1.026 (0.981-1.074) | 0.263 | 0.026 | - | - | - |
| NHHR | 1.187 (1.108-1.272) | < 0.001* | 0.172 | 1.147 (1.056-1.246) | 0.001* | 0.137 |

Abbreviations: BMI, body mass index; MAP, mean arterial pressure; PCI, percutaneous coronary intervention; CABG, coronary artery bypass grafting; BNP, brain natriuretic peptide; HbA1c, glycated hemoglobin A1c; FBG, fasting blood glucose; hs-CRP, high sensitivity C-reactive protein; RBC-CV, red blood cell distribution width coefficient of variation; PDW, platelet distribution width; PCSK9i, presenilin converting enzyme kexin-9 inhibitor; SGLT2i, sodium-dependent glucose transporters 2 inhibitors; GLP-1RA, glucagon-like peptide-1 receptor agonists; LVEF, left ventricular ejection fraction; LVEDD, left ventricular end-diastolic dimension; LDL-C, low-density lipoprotein cholesterol; HDL-C, high-density lipoprotein cholesterol; TG, triglyceride; NHHR, Non-High-Density Lipoprotein Cholesterol to High-Density Lipoprotein Cholesterol Ratio. *<0.05

**Supplementary Table 4. Collinearity analysis of risk factors with NHHR**

| **Variables** | **Standardized coefficient** | | **Covariance analysis** | |
| --- | --- | --- | --- | --- |
|  | ***B*** | ***P*** | ***Tolerance*** | ***VIF*** |
| Age (year) | 0.092 | <0.001* | 0.728 | 1.373 |
| Male, n (%) | 0.032 | 0.181 | 0.806 | 1.241 |
| BMI (kg/m2) | 0.013 | 0.576 | 0.879 | 1.138 |
| CABG history | 0.088 | <0.001* | 0.954 | 1.048 |
| Type 2 Diabetes | 0.049 | 0.079 | 0.591 | 1.691 |
| Heart failure | -0.013 | 0.568 | 0.832 | 1.201 |
| Cerebrovascular disease | 0.041 | 0.060 | 0.948 | 1.054 |
| PCI | 0.144 | <0.001* | 0.817 | 1.224 |
| PTCA | 0.127 | <0.001* | 0.900 | 1.111 |
| Aspirin | 0.038 | 0.094 | 0.901 | 1.110 |
| Ticagrelor | 0.072 | 0.003* | 0.777 | 1.287 |
| D-Dimer (mg/L) | -0.016 | 0.497 | 0.849 | 1.177 |
| BNP (pg/mL) | 0.069 | 0.020* | 0.532 | 1.909 |
| Creatinine (umol/L) | -0.020 | 0.411 | 0.752 | 1.305 |
| HbA1c (%) | -0.045 | 0.181 | 0.407 | 2.013 |
| FBG (mmol/L) | 0.063 | 0.040* | 0.497 | 2.051 |
| Albumin (g/L) | 0.044 | 0.087 | 0.708 | 1.412 |
| hs-CRP (mg/L) | 0.036 | 0.143 | 0.764 | 1.309 |
| LVEF (%) | -0.210 | <0.001* | 0.667 | 1.628 |
| LVEDD (mm) | 0.020 | 0.908 | 0.920 | 1.091 |
| ApolipoproteinA1 (mmol/L) | -0.089 | 0.032* | 0.266 | 3.761 |
| Apolipoprotein B (mmol/L) | -0.016 | 0.808 | 0.108 | 9.246 |
| Lipoprotein (a) (mmol/L) | 0.042 | 0.058 | 0.933 | 1.091 |
| LDL-C (mmol/L) | 0.072 | 0.196 | 0.150 | 6.670 |
| Total cholesterol (mmol/L) | 0.071 | 0.204 | 0.148 | 6.765 |
| Apolipoprotein B/A1 | -0.034 | 0.530 | 0.155 | 6.437 |

Abbreviations: BMI, body mass index; PCI, percutaneous coronary intervention; CABG, coronary artery bypass grafting; BNP, brain natriuretic peptide; HbA1c, glycated hemoglobin A1c; FBG, fasting blood glucose; hs-CRP, high sensitivity C-reactive protein; LVEF, left ventricular ejection fraction; LVEDD, left ventricular end-diastolic dimension; LDL-C, low-density lipoprotein cholesterol. *<0.05

**Supplementary Table 5. Subgroups analyze**

|  | | **High Gensini score** | | | | | |
| --- | --- | --- | --- | --- | --- | --- | --- |
|  |  | ***OR (95% CI)*** | ***P*** |  | ***OR (95% CI)*** | ***P*** | ***P for interaction*** |
| Age≥65years | | |  | Age<65years | |  | 0.407 |
| NHHR | Q1 | *Reference* | 0.149 | Q1 | *Reference* | 0.013 |  |
|  | Q2 | 0.927 (0.638-1.348) | 0.692 | Q2 | 1.927 (1.151-3.225) | 0.013 |  |
|  | Q3 | 1.231 (0.840-1.805) | 0.287 | Q3 | 1.769 (1.060-2.950) | 0.029 |  |
|  | Q4 | 1.457 (0.942-2.255) | 0.091 | Q4 | 2.313 (1.388-3.855) | 0.001 |  |
| Male | | |  | Female | |  | 0.302 |
| NHHR | Q1 | *Reference* | 0.057 | Q1 | *Reference* | 0.173 |  |
|  | Q2 | 1.120 (0.791-1.588) | 0.523 | Q2 | 1.172 (0.649-2.117) | 0.599 |  |
|  | Q3 | 1.465 (1.028-2.086) | 0.034 | Q3 | 0.935 (0.503-1.736) | 0.831 |  |
|  | Q4 | 1.553 (1.065-2.262) | 0.022 | Q4 | 1.816 (0.952-3.463) | 0.070 |  |
| BMI≥28kg/m^2^ | | |  | BMI<28kg/m^2^ | |  | 0.937 |
| NHHR | Q1 | *Reference* | 0.389 | Q1 | *Reference* | 0.040 |  |
|  | Q2 | 1.335 (0.618-2.884) | 0.462 | Q2 | 1.165 (0.840-1.617) | 0.361 |  |
|  | Q3 | 2.072 (0.982-4.372) | 0.056 | Q3 | 1.326 (0.949-18.54) | 0.098 |  |
|  | Q4 | 2.428 (1.134-5.201) | 0.022 | Q4 | 1.670 (1.164-2.397) | 0.005 |  |
| CABG/PCI history | | |  | No-CABG/PCI history | |  | 0.544 |
| NHHR | Q1 | *Reference* | 0.550 | Q1 | *Reference* | 0.025 |  |
|  | Q2 | 1.583 (0.830-3.021) | 0.163 | Q2 | 1.025 (0.735-1.429) | 0.885 |  |
|  | Q3 | 1.408 (0.717-2.762) | 0.320 | Q3 | 1.299 (0.931-1.813) | 0.124 |  |
|  | Q4 | 1.252 (0.585-2.679) | 0.562 | Q4 | 1.595 (1.122-2.267) | 0.009 |  |
| Lp(a)≥125nmol/L | | |  | Lp(a)<125nmol/L | |  | 0.462 |
| NHHR | Q1 | *Reference* | 0.030 | Q1 | *Reference* | 0.413 |  |
|  | Q2 | 1.102 (0.737-1.649) | 0.636 | Q2 | 1.180 (0.763-1.824) | 0.457 |  |
|  | Q3 | 1.244 (0.830-1.862) | 0.290 | Q3 | 1.450 (0.927-2.268) | 0.104 |  |
|  | Q4 | 1.785 (1.171-2.721) | 0.007 | Q4 | 1.348 (0.819-2.217) | 0.240 |  |
| UA | | |  | STEMI/USTEMI | |  | 0.582 |
|  | Q1 | *Reference* | 0.108 | Q1 | *Reference* | 0.165 |  |
|  | Q2 | 1.303 (0.757-2.241) | 0.339 | Q2 | 1.082 (0.749-1.564) | 0.675 |  |
|  | Q3 | 1.983 (1.089-3.612) | 0.025 | Q3 | 1.161 (0.809-1.667) | 0.417 |  |
|  | Q4 | 1.884 (0.941-3.770) | 0.074 | Q4 | 1.486 (1.018-2.170) | 0.040 |  |

Abbreviations: BMI, body mass index; PCI, percutaneous coronary intervention; CABG, coronary artery bypass grafting; NHHR, Non-High-Density Lipoprotein Cholesterol to High-Density Lipoprotein Cholesterol Ratio; GS, Gensini score; Lp(a), Lipoprotein (a); UA, unstable angina; STEMI, ST-segment elevation myocardial infarction, NSTEMI, Non-ST-segment elevation myocardial infarction; OR, odds ratio.


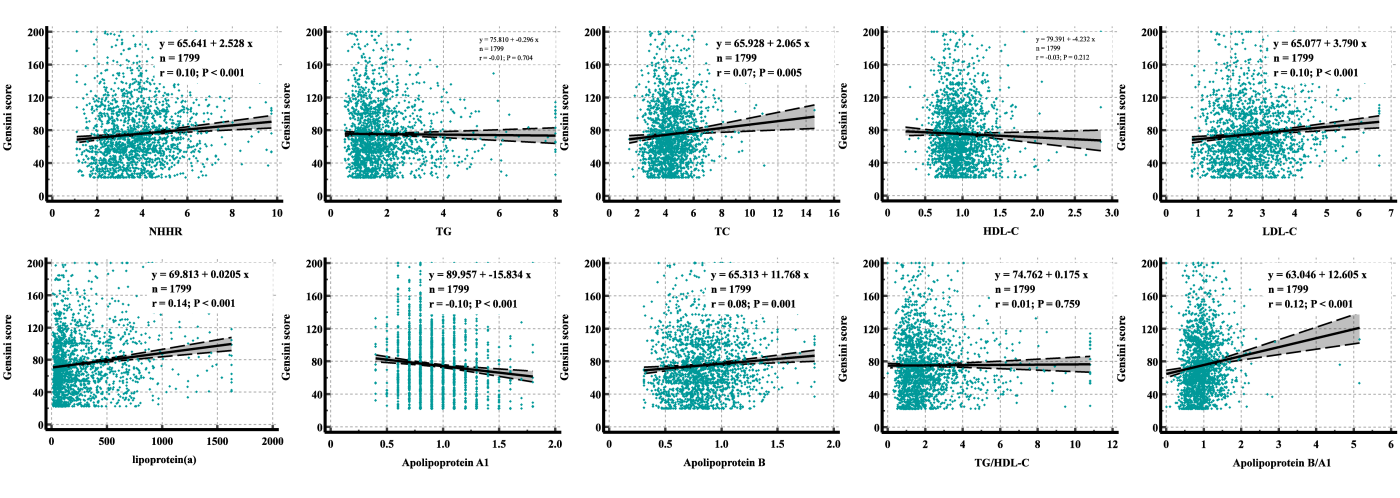


**Supplementary Figure 1.** **Correlation between NHHR and different blood lipids.** Abbreviations: NHHR, Non-High-Density Lipoprotein Cholesterol to High-Density Lipoprotein Cholesterol Ratio; LDL-C, low-density lipoprotein cholesterol; HDL-C, high-density lipoprotein cholesterol; TC, triglyceride; TG, triglyceride.


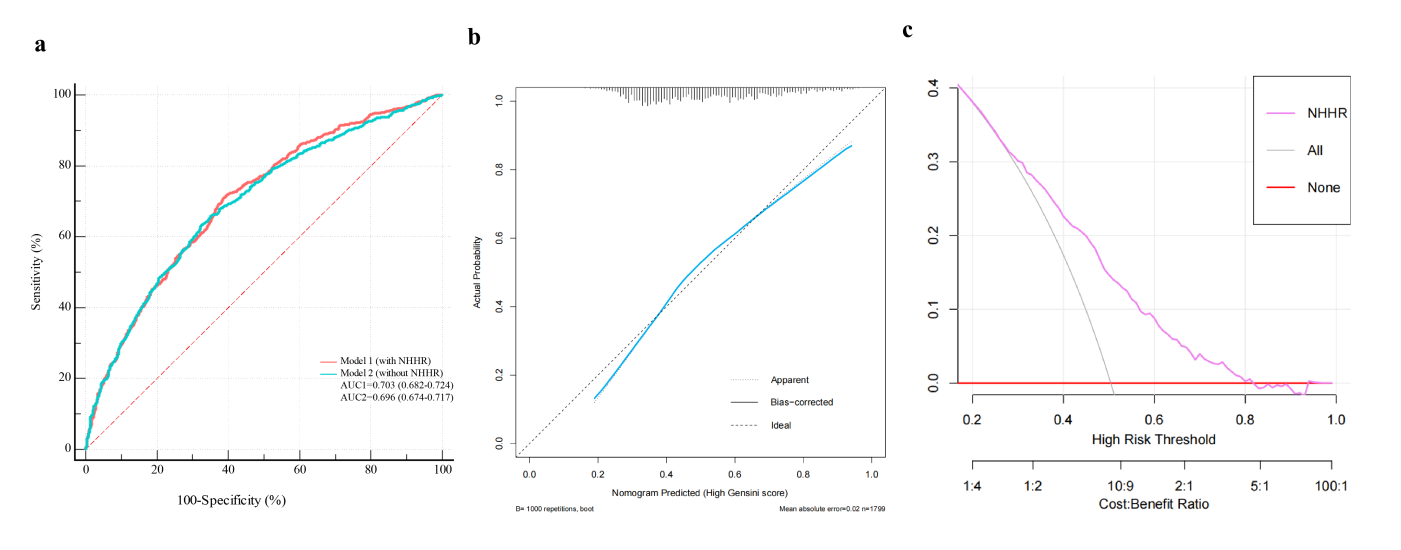


**Supplementary Figure 2. The ROC curve of model 1 and model 2 for predicting high** **Gensini score (a) and calibration plots (b) and decision curve analysis (c) of Model 1.** Abbreviations: NHHR, Non-High-Density Lipoprotein Cholesterol to High-Density Lipoprotein Cholesterol Ratio; AUC: area under the curve.
